# Expert-opinion-based guidance for the care of children with lysosomal storage diseases during the COVID-19 pandemic: An experience-based Turkey perspective

**DOI:** 10.3389/fpubh.2023.1092895

**Published:** 2023-01-30

**Authors:** Abdurrahman Akgun, Gulden Gokcay, Neslihan Onenli Mungan, Hatice Serap Sivri, Hasan Tezer, Cigdem Aktuglu Zeybek, Fatih Ezgu

**Affiliations:** ^1^Division of Pediatric Metabolism, Department of Pediatrics, Faculty of Medicine, Firat University, Elazig, Turkey; ^2^Division of Nutrition and Metabolism, Department of Pediatrics, Faculty of Medicine, Istanbul University, Istanbul, Turkey; ^3^Division of Pediatric Metabolism, Department of Pediatrics, Faculty of Medicine, Cukurova University, Adana, Turkey; ^4^Division of Pediatric Metabolism, Department of Pediatrics, Faculty of Medicine, Hacettepe University, Ankara, Turkey; ^5^Department of Infectious Diseases, Faculty of Medicine, Gazi University, Ankara, Turkey; ^6^Division of Pediatric Metabolism, Department of Pediatrics, Faculty of Medicine, Istanbul University Cerrahpasa, Istanbul, Turkey; ^7^Division of Pediatric Metabolism and Division of Pediatric Genetics, Department of Pediatrics, Faculty of Medicine, Gazi University, Ankara, Turkey

**Keywords:** lysosomal storage diseases, COVID-19 pandemic, specialized care, expert opinion, immune-inflammatory mechanisms

## Abstract

This expert-opinion-based document was prepared by a group of specialists in pediatric inherited metabolic diseases and infectious diseases including administrative board members of Turkish Society for Pediatric Nutrition and Metabolism to provide guidance for the care of children with lysosomal storage disorders (LSDs) during the COVID-19 pandemic in Turkey. The experts reached consensus on key areas of focus regarding COVID-19-based risk status in relation to intersecting immune-inflammatory mechanisms and disease patterns in children with LSDs, diagnostic virus testing, particularly preventive measures and priorities during the pandemic, routine screening and diagnostic interventions for LSDs, psychological and socioeconomic impact of confinement measures and quarantines and optimal practice patterns in managing LSDs and/or COVID-19. The participating experts agreed on the intersecting characteristics of immune-inflammatory mechanisms, end-organ damage and prognostic biomarkers in LSD and COVID-19 populations, emphasizing the likelihood of enhanced clinical care when their interaction is clarified *via* further studies addressing certain aspects related to immunity, lysosomal dysfunction and disease pathogenesis. In the context of the current global COVID-19 pandemic, this expert-opinion-based document provides guidance for the care of children with LSDs during the COVID-19 pandemic based on the recent experience in Turkey.

## Introduction

The initial reports during the early phase of coronavirus disease 2019 (COVID-19) indicated a milder course in the pediatric age with progression to severe or critical respiratory distress only in 2–6% of infected cases (1), as suggested to be related to the high plasticity of the immune system, low expression of ACE2 receptors or the frequent exposure to other coronaviruses in the pediatric age group (2–4). Direct virus-virus interactions occur due to the presence of other simultaneous viruses in the lower respiratory mucosa in children, which can lead to competition and limit the viremia of SARS CoV-2 (5).

However, starting from mid-April 2020, clusters of pediatric cases epidemiologically linked with COVID-19, mediated by a post-infectious inflammatory response following SARS-CoV-2 rather than a direct viral invasion, have been reported as presenting with fever, hypotension, predominance of gastrointestinal complications and cardiac dysfunction (6). Centers for Disease Control and Prevention (CDC), and the World Health Organization (WHO) named this new hyper-inflammatory syndrome emerged in older school-aged children and adolescents as SARS-CoV-2-associated multisystem inflammatory syndrome in children (MIS-C) (7, 8).

Accordingly, the current status of pediatric COVID-19 is sufficient to alert pediatricians (9), given the risk of overlooking the possibility of developing a severe infection in children with underlying/comorbid diseases (10). Despite their heterogeneity, rare diseases share certain features such as being chronic, complex, progressive and severely disabling diseases that are in need of specific care (11), while the treatment is directed only by a limited number of referral centers due to scarcity of expertise to provide the best quality care (11–16). Overall, ~ 350 million people worldwide are estimated to suffer from a rare disease, while in Turkey, due to higher frequency of consanguineous marriages (24%), the rare diseases are considered to be more prevalent than in other Western countries, probably affecting ~ 5 to 7 million people (17, 18).

Lysosomal storage disorders (LSDs) are a group of inherited metabolic diseases secondary to lysosomal enzyme defects and are characterized by a progressive accumulation of non-digested macromolecules provoking end-organ damage leading to severe multisystem disease and premature death (19, 20). LSD-specific treatments, consisting of enzyme replacement therapy (ERT), or oral medications (substrate reduction therapy, chaperones) are available and require regular administration to be effective (21, 22).

Children with LSDs refer to a special population not only in terms of unique challenges in the provision of specialized care during the pandemic but also might bear the risk of having more severe progression during a possible COVID-19 disease, given that they are susceptible to impaired autophagy, hyper-inflammation, and possibly to reduced infection control (23).

Given the expected increase in the number of COVID-19 infected children as the pandemic continues to spread (24), the potential social and economic consequences of the pandemic are likely to limit the access of patients to critical healthcare resources (23). Accordingly, developing guidelines on managing children with COVID-19 during the pandemic, with a particular emphasis on high-risk pediatric populations with pre-existing chronic conditions, is considered crucial to provide the best care for vulnerable children and to minimize their risk of progression to critical disease or death (24).

This expert-opinion-based document was therefore prepared by a group of specialists in pediatric inherited metabolic diseases and infectious diseases including administrative board members of Turkish Society for Pediatric Nutrition and Metabolism to provide guidance for the care of children with LSDs (Gaucher disease, Fabry disease, Mucopolysaccharidosis, and Pompe disease) during the COVID-19 pandemic based on the recent experience in Turkey.

Supported by the available scientific evidence and expert clinical opinion, a number of key questions were addressed in this consensus document, regarding the COVID-19 based risk status in children with LSDs in relation to immune-inflammatory mechanisms and disease patterns, diagnostic virus testing, particular preventive measures and priorities during the pandemic, difficulties in ongoing treatment protocols such as ERTs, routine screening and diagnostic interventions for LSDs, psychological and socioeconomic impact of preventive measures and quarantines and optimal practice patterns in managing LSDs during COVID-19 pandemic.

## Should children with LSD be considered at risk of increased susceptibility to SARS-CoV-2 infection and severe COVID-19 based on immune-inflammatory mechanisms and disease patterns?

### The immune-inflammatory mechanisms in COVID-19 and lysosomal diseases

Although the exact pathophysiology of COVID-19 still remains unknown, the potential role of multiple cytokines and/or anti-cytokine interventions has become clearer with growing scientific data, indicating the contribution of both immunodeficiency and hyper-inflammation (cytokine storm) in the pathogenesis of disease (25).

**Figure 1:**
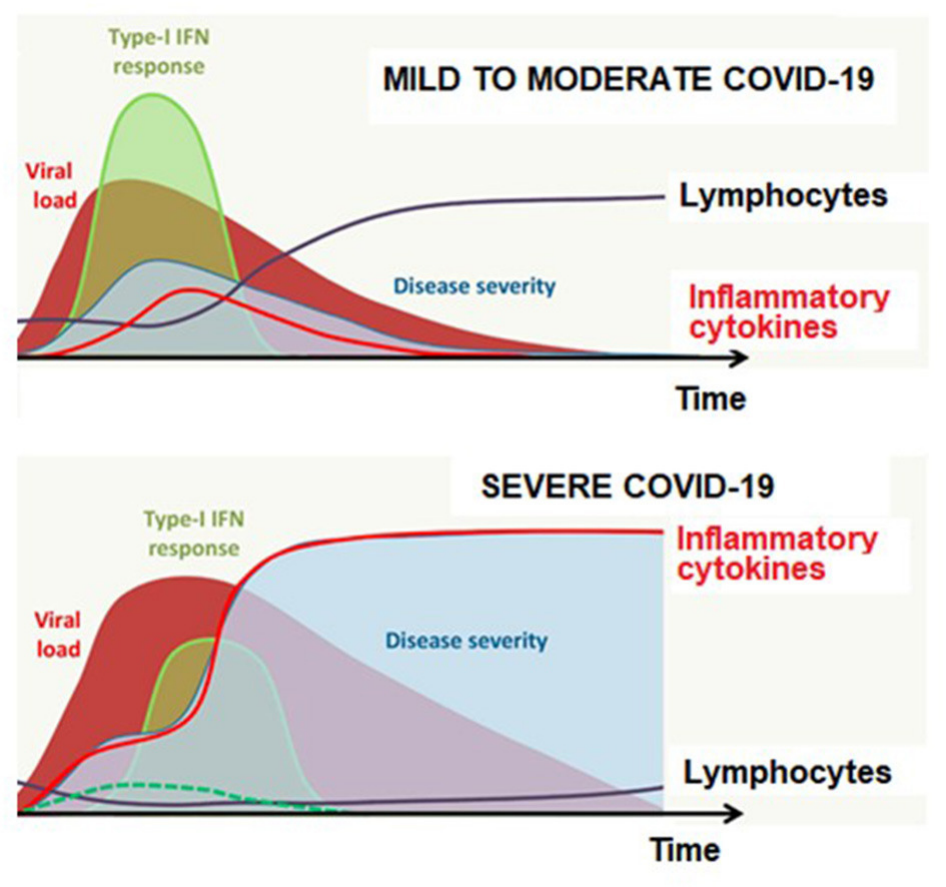
Antiviral response according to disease severity and time in COVID-19. Adapted from Jamilloux et al. (25).

Cytokines seem to be central to the pathophysiology of COVID-19, playing either a beneficial (type-I interferon, IL-7) or a detrimental (IL-1β, IL-6, and TNF-α) role particularly in the context of cytokine storm, while the role of concomitant immunodeficiency is also noted involving impaired type-I interferon response and lymphopenia (25) (Figure 1).

In accordance with current knowledge on the role of cytokine storm in other respiratory infections, combined antiviral treatments, and targeted immunosuppression has been offered as a therapeutic strategy in COVID-19 (26). There are now multiple clinical trials testing the impact of targeted immune-suppressants (26, 27).

SARS-CoV-2 uses the lysosomal/endosomal system to infect cells (23). Healthy lysosomes are essential for developing normal host response to infection and maintaining a normal inflammatory response given their role in the regulation of autophagy, control of inflammasome release of cytokines, and the regulation of sphingolipid metabolism (28). Hence patients with LSDs are susceptible to impaired autophagy, hyper-inflammation, and possibly to reduced infection control not only due to defective lysosomes but also substrate storage-dependent activation of inflammatory pathways (29).

In addition, defective lysosomes are also associated with disturbed energy balance and mitochondrial metabolism to provide ATP for survival (29, 30). Notably, cellular ATP (cATP) depletion has been proposed to be a crucial component in the infectivity and prognosis of COVID-19. Thus, an increase in c-ATP (ATP depletion) is suggested to ameliorate immune dysregulation through activation of initial IFN-1 secretion and signaling, as “initial alarm” of the innate immune system and through the prevention of “cytokine storm” and T-cell apoptosis (31).

Specifically, in Gaucher disease, the accumulation of glucosylceramide and glucosylsphingosine in macrophage leads to macrophage activation-mediated release of various cytokines (i.e., chitotriosidase, TNF-a, IL-1b) (28), while there is also lysosomal dysfunction-mediated trigger of auto-inflammatory cascades involving a broad spectrum of myeloid cells, cytokine/chemokine secretion and NLRP3 inflammasome activation (23, 32). Polyclonal and monoclonal gammopathies were also reported in Gaucher disease in addition to thrombocytopenia, disturbed hemostasis and spleen infarcts (28, 33–35).

Notably, elevated cytokines found in SARS-CoV-2 (IL-2R, IL-6, IL-10, MIP1-α, and TNF-α), are reminiscent of the pattern described in Gaucher disease (23, 36, 37), and although generally macrophages are not considered to be direct targets of the SARS-CoV-2 virus, preliminary evidence from human autopsy material indicated that ACE2-expressing CD169+ macrophages in the spleen and lymph nodes to be positive for SARS-CoV-2 nucleoprotein antigen, suggesting their contribution to viral spread and inflammation in SARS-CoV-2 infection (38). Moreover, the immunopathology of lung injury in ARDS, the most advanced form of SARS-CoV-2 infection, involves a central role for myeloid cells (23).

Indeed, owing to its primary role in triggering the cytokine storm in COVID-19, the higher plasma level of IL-6 is considered as a significant marker of worse disease prognosis in COVID-19 infected patients (39–41). Elevated IL-6 levels were also reported in LSDs, including patients with Gaucher disease (associated with the expansion of myeloid cells) (28) and those MPS IVA patients undergoing ERT (combined with induced pro-oxidant states) (42).

Elevated ferritin levels (suggestive of hyper-inflammation), lymphopenia and higher levels of D-dimer (a marker of a hypercoagulable state and endogenous fibrinolysis) were associated with disease severity in COVD-19 patients (43–45). This seems notable given that D-dimer levels were reported to be significantly elevated in Gaucher disease and considered as a potential marker in risk prediction of bone and lung involvement as well as in the assessment of treatment response (46). In addition to macrophage-derived cytokines and proteins, increased concentrations of ACE and plasma ferritin are also hallmark biomarkers for Gaucher disease and known to decrease in response to therapy (47, 48).

Another LSD with a major role of inflammation and an abnormal immune response is Fabry disease, in which the globotriaosylceramide (Gb3) is the accumulated glycosphingolipid, due to the deficiency of the enzyme a-galactosidase A (49). Deficiency of this enzyme in Fabry disease causes aberrant accumulation of lipid antigens and activation of immature CD1d-restricted natural killer T (NKT) cells, resulting in autoimmunity (49). The pro-inflammatory feature of Fabry disease also has been well described, with TLR4 pathway-dependent increase in inflammatory cytokines including IL-6, IL-1b, and TNF-a (50).

The TLR4 pathway is also activated in mucopolysaccharidosis (MPS), parallel to the accumulation of glycosaminoglycans (GAGs) (28). GAG storage in MPS leads to TLR4-mediated elevation in pro-inflammatory cytokines, predominantly TNF-a and IL-1b (28, 51). Hence, both in Fabry disease and MPS substrate accumulation is considered to trigger TLR4 and CD1d systems contributing to irreversible organ damage (49, 52).

**Figure 2:**
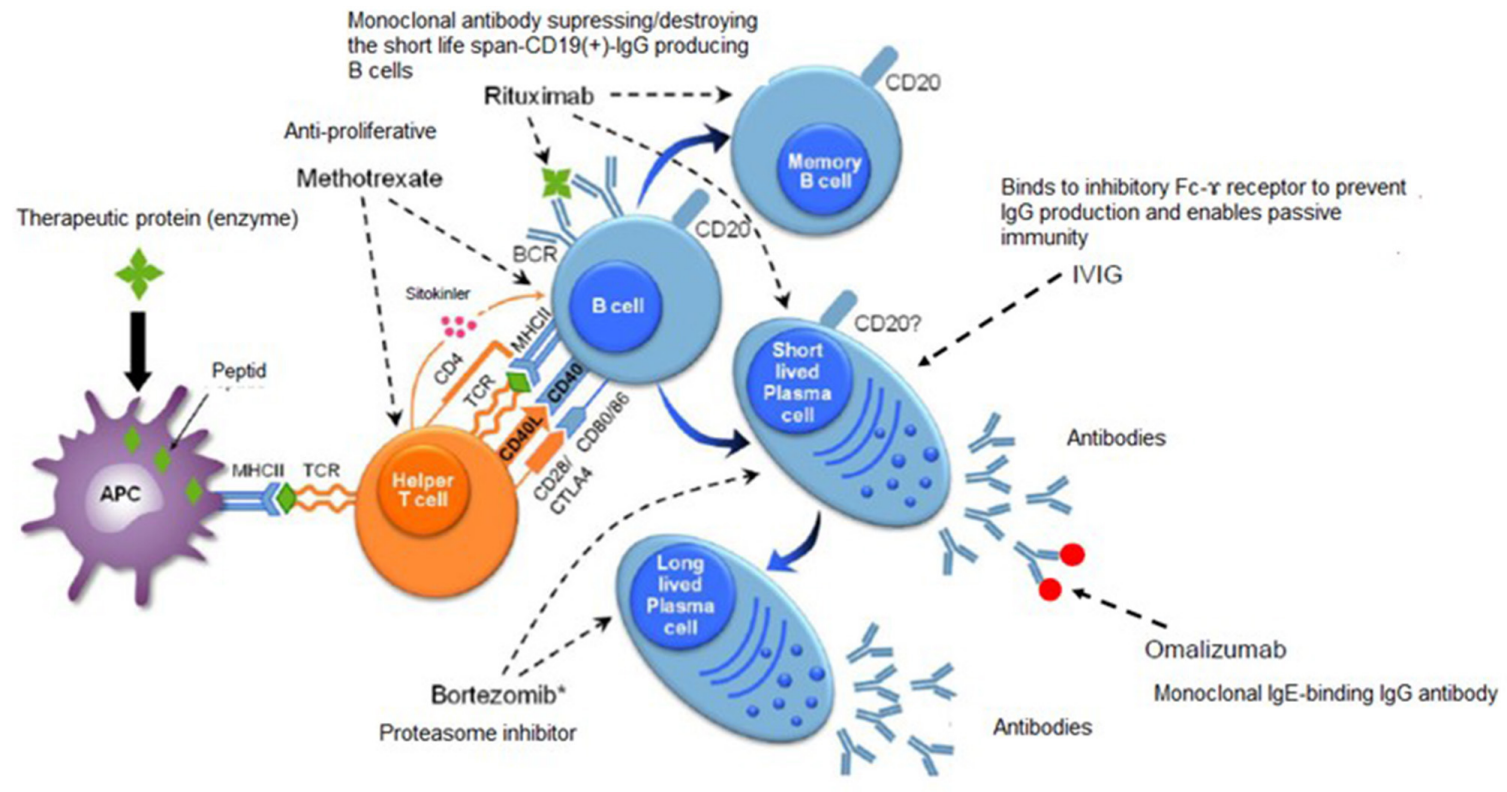
Immunomodulatory treatments in Pompe disease. Adapted from Kishnani et al. (53).

In Pompe disease, characterized by massive glycogen deposition in skeletal, cardiac and smooth muscle secondary to the deficiency of acid α-glucosidase (GAA), development of an immune response with resultant loss of therapeutic efficacy of ERT remains a significant challenge, particularly in the infantile form (53, 54). Accordingly, several immunomodulatory protocols, just as those used to treat COVID-19 patients, have been applied to optimize therapeutic efficacy of ERT for Pompe, including various combinations of rituximab (anti-CD20 monoclonal antibody targets CD20-positive B cells), bortezomib (a proteasome inhibitor, targets mature antibody-producing plasma cells), intravenous immunoglobulin (IVIg, downregulates antibody responses by binding to inhibitory FcR and also protects against infectious agents in immune-suppressed conditions), cyclophosphamide, methotrexate (targets both B and T cells) especially in patients who lack cross reacting immune material (CRIM) (53) (Figure 2).

### The overlapping characteristics of end-organ damage

In addition, while certainly needs to be justified by further investigation, the end-organ damage characteristics provided in Table 1 suggests to indicate the likelihood of COVID-19 as well as MIS-C to share certain disease pathways (i.e. pulmonary system including airways, cardiovascular and nervous system) with Gaucher disease (plus disturbed hemostasis and gastrointestinal involvement), Fabry disease (plus renal and gastrointestinal involvement), MPS and Pompe disease, beyond similarities in the pattern of hypercytokinemia (6, 23, 55–58). This would not only complicate the follow-up of the patients with an add-on COVID-19 in terms of organ involvement but also might complicate the diagnostic process during the pandemic.

**Table 1:** End-organ damage in COVID-19 and lysosomal storage diseases (23, 55–57).

	**COVID-19**	**Gaucher disease**	**Fabry disease**	**MPS**	**Pompe disease**
**Airways, lungs**	Pneumonia ARDS DIC	CPA Pulmonary hypertension Alveolar hypoventilation	Obstructive disease ↓ diffusion capacity	Alveolar hypoventilation	LRTI Alveolar hypoventilation
**Cardiovascular system**	Inflammation Rhythm disorder Heart failure Infarcts	Valvular disease Calcifications	Rhythm disorder Low diastolic BP Cardiomyopathy Increased CIMT	Cardiomyopathy Valvular disease Increased CIMT	Cardiomyopathy
**Nervous system**	Encephalitis ANHE	Type 2: Severe Type 3: ANP	TIA Syncope	Mental-motor retardation	Myopathy (adults)
**Kidney**	Hematuria Proteinuria ARF	-	Proteinuria CRF	-	**-**
**GIS and liver**	Vomiting Diarrhea AVH	Dyspepsia Distension Reflux	Vomiting Diarrhea Abdominal pain	Organomegaly	**-**
**Hemostasis**	DIC VTE	Thrombocytopenia ↓Factor concentration	-	-	**-**

ANHE, Acute necrotising hemorrhagic encephalopathy; ANP, Abducens nerve palsy; ARDS, Acute respiratory distress syndrome; ARF, Acute renal failure; AVH, Acute viral hepatitis; BP, Blood pressure; CIMT, Carotid intima-media thickness; CRF, Chronic renal failure; CPA, Chronic pulmonary aspiration; DIC, Disseminated intravascular coagulation, GIS, Gastrointestinal system; LRTI, Lower respiratory tract infections; MPS, Mucopolysaccharidosis; TIA, Transient ischemic attack; VTE, Venous thromboembolism. ↓Decreased.

#### Consensus statements on COVID-19-based risk status in children with LSDs

Although any infection would create a risk for complicating the clinical course of untreated or complicated LSD, this risk might not be significantly increased by COVID-19 in especially treated patients. An exception would be the infantile and adult forms of Pompe disease in which direct involvement of the heart and the lungs in the infantile form and indirect involvement of the lungs due to the involvement of the respiratory muscles in the adult form might result in a more severe clinical course with add-on COVID-19 even during treatment. Also, patients with symptomatic Fabry related cardiomyopathy bear increased risk with add-on COVID-19 or MIS-C. More definite and evidence-based assumptions can be made after retrospective evaluation of the patient outcomes during the pandemic period.

The reorganization of the outpatient clinics, as well as the infusion centers in addition to the fear of getting COVID-19 during the visits, seems to be the major obstacles for the regular visits and treatment of patients with LSDs. Also, some patients have to travel to other cities due to the lack of metabolic disease centers in their home towns and travel restrictions as well as the reorganization of the hospitals as pandemic centers provide a significant obstacle for the maintenance of their healthcare especially for enzyme replacement therapy.

While objective data is limited, based on the personal experience of the board members, the number of patients infected with SARS-CoV-2 was not significantly higher compared to normal population. This may be due to the particular attention of these patients/caregivers in respecting measures of hygiene and infection prevention.

Accordingly, in children with LSDs who presented with COVID-19 symptoms, the following approaches should be implemented:

- Initial evaluation should involve sequential inflammatory markers, including CBC/differential, CRP, erythrocyte sedimentation rate; coagulation parameters including D-dimer and ferritin; liver function markers; and a cytokine panel.
- Those without end-organ involvement and no signs of cardiac, respiratory of hematological deterioration should still be considered at risk, and close follow-up should be applied after diagnostic virus testing.
- Those with end-organ involvement indicating cardiac, pulmonary or hematological disorders should be considered at high risk and hospitalized and treated with close monitoring after virus diagnostic testing. However, given the absence of COVID-19 specific treatment and the potential adverse effects of agents used in current empirical treatment protocols in children with LSD, the possible drug-drug interactions should carefully be considered in the decision for the treatment.

Children with LSDs are not considered among the high-risk vulnerable populations or for priority diagnostic testing for COVID-19 (PCR) in general and the national routine testing indications could also well be valid for patients with LSDs.

Still, it is imperative to collect epidemiologic data regarding exposures and positive infection rates to understand the natural course of COVID-19 and risk factors for severe disease in children with LSDs. Accordingly, a nationwide cross-sectional screening study should be planned in children with metabolic disorders to address the rate of herd immunity (after availability of specific validated antibodies tests) and active infection (PCR-based screening) to evaluate the clinical course of COVID-19 in subgroups of children with acute metabolic decompensation vs. LSDs and in those staying at home vs. receiving in-hospital ERT.

## Should specific criteria be set in implementation and interpretation of diagnostic virus testing among children with LSDs?

### Consensus statements on virus testing

No special implementation (PCR vs. antibody) or interpretation criteria are required for COVID-19 diagnostic testing in children with LSD.

However, appropriate collection of samples with a correct implementation of oropharyngeal and nasopharyngeal swabs seems crucial to reveal accurate results similar to patients without LSDs.

In addition, recent emerge of MIS-C in children as a syndrome temporally associated with COVID-19 is important given that it manifests with persistent fever, cytokine storm, coagulopathies, cardiac dysfunction, gastrointestinal symptoms and acute kidney injury rather than respiratory symptoms as prominent features, requiring hospitalization in many of children and progressing to a shock-like clinical picture in some of them (6, 59, 60). This clinical syndrome can also occur in children with LSDs and, it is recommended to include antibody testing in addition to PCR testing for SARS-CoV-2, given that some children are antibody-positive even when PCR-negative, suggesting that inflammatory complications were delayed, occurring when the virus was no longer detectable on nasal swabs (60).

## Are there specific measures or priorities to protect children with LSDs from COVID-19 infection?

EURORDIS states that children with rare diseases should have regular access to medical care with no restriction or disruption in the continuity of care, including routine treatments (i.e., infusions) administered in the hospital setting. Accordingly, given that LSDs diseases can be highly debilitating and life-threatening, canceled or postponed routine medical interventions or travel restrictions bears the potential of increased risk of deterioration of symptoms and future burden on healthcare systems due to possible complications (11). In addition, people living with LSDs may put themselves at higher risk by staying at home due to considerable barriers they face in receiving health care in the hospital setting such as lack of protocols set in place for their care during pandemic and fear of visiting hospitals because of the risk of catching SARS-CoV-2 infection (11).

### Consensus statements on protective measures and priorities during the pandemic

Children with LSDs should be given moderate priority, without increasing the risk of discrimination and stigma, in access to preventative measures (i.e., mask, testing, and access to personal protective equipment), given they need routine treatment administration that occurs in the hospital setting and feels anxiety regarding the risk of being contaminated during travel or in-hospital procedures.

Availability of SARS-CoV-2 free hospitals for children with LSDs to receive routine treatments without encountering infected patients, and creation of temporary special hospital wards for those affected by COVID-19 is critical. Until this is available, suspected or confirmed COVID-19 patients must be separated from non-COVID-19 LSD patients by clear physical measures (i.e., in separate buildings, separate groups of healthcare workers) within the same hospital/campus if the health facility has to look after both groups of patients. Also reinforcing hygiene rules and applying barrier measures among both groups of patients and healthcare professionals who would take care of the patients are very important.

Developing and adopting concrete protocols is necessary to standardize the clinical practice and to meet the complex needs of children with LSDs in the provision of healthcare during the COVID-19 crisis.

During the pandemic period it would be useful to supply the patients/parents with relevant and directing information (i.e., rules of hygiene, contact details about the centers with active infusion units) with tools such as FAQ sheet shared on the society website, advice hotlines by the reference centers, patient brochures, and social media posts updated in accordance with increased knowledge to keep them informed and safe.

With the recent availability of vaccines toward SARS-CoV-2, patients with LSDs should always be encouraged to be vaccinated. The potential harm that could result from use of “live attenuated” vaccines especially for patents with Pompe Disease receiving immunomodulation should be considered.

## What are the implications of current pandemic on routine screening and diagnosis of LSDs?

Screening and confirmatory tests such as enzyme and molecular assays carry significant importance for diagnosis and early initiation of treatment. Accordingly, not being able to perform them during the pandemic is considered detrimental to the health of those who are yet undiagnosed, as this would result in delayed initiation of treatment (11).

### Consensus statements on routine screening and diagnosis interventions during the pandemic

It is out of the question to delay screening and diagnosis of LSDs in a potential patient during the time current crisis. Accordingly, maintenance of even minimum screening service is crucial to ensure the timely diagnosis as well as testing for conditions likely to deteriorate in formerly diagnosed patients. This can be achieved by adopting SARS-CoV-2 free hospital policy with pre-triage algorithm and referral of suspected cases to pandemic units.

If the patient/parents are hesitant to come to the visit and/or the clinic center is not available, then teleconsultations or even e-mail based consultations should be considered. However, the Ministry of Health should form the regulatory basis of online outpatient appointments, teleconsultations or e-mail correspondence. These types of “remote” contacts would also facilitate the “refilling” of the prescriptions and patient reimbursement reports with less hesitation.

Although the creation of “pandemic” hospitals and “SARS-CoV-2 free” hospitals is critical to prevent the spread of COVID-19 within different groups of patients, this was not able to be put into practice in Turkey. The majority of the hospitals bearing metabolic centers were announced as pandemic hospitals. Despite the fact that COVID-19 and metabolic units were reliably separated in terms of physical localization and healthcare personnel, it should be noted that there is considerable amount canceled or postponed appointments in the current clinical practice at patient's discretion in most cases or due to inability to provide enough outpatient healthcare in some hospitals. A significant increase in patient load was reached creating difficulty in managing the patients during normalization. Special precautions should be considered for future normalization periods after close-ups.

## What are the psychological and socioeconomic impact of confinement measures and quarantines in LSD community?

The implementation of confinement measures and quarantines to avoid the spread of COVID-19 can have a severe impact on the provision of holistic care for people living with a rare disease. Alongside the routine at-hospital follow-up visits, the sustainability of resource centers that offer rehabilitation therapy, physiotherapy, respiratory care and daycare is also disrupted given they close entirely or reduce their capacity for services (11). Confinement measures can have a severe psychological impact on people living with a rare disease due to the isolation and exacerbate their existing psychological problems since their unhappiness and depression rates are already higher than the general population under normal circumstances (11). In addition, confinement can be very stressful for both the patient and caregivers, given that in certain groups of these patients, outdoor activity or socialization and schooling are part of the therapeutic routine (11).

Moreover, the inappropriate social environment could increase the risk of infection and adverse COVID-19 outcomes due to poor socioeconomic status (poor, large families or crowded housing) and unhealthy geographic area (polluted environments or in areas without reliable access to clean water) (61).

### Consensus statements on the psychological and socioeconomic impact of confinement measures and quarantines

Continuity of social care and support service is particularly important in children with LSDs during the time of crisis, while patient organizations are also crucial in terms of their role in enabling information flows with healthcare services, providing peer support and developing creative solutions (11).

Given the risk of confinement measures to deteriorate behavioral functions, children with LSDs should be given special dispensation allowing outdoor activities with a caregiver during lockdown periods as long as hygienic and security measures are followed.

The closure of specialized child care facilities and schools means that families of children with LSD are unable to work and obliged to take full-time responsibilities of care for their children. Hence, schools or specialized care facilities should take measures to guarantee continued education from home for students living with LSDs and should be in close relation with family members to help them to balance their work and care duties.

Parents of children with LSDs should be offered special accommodations by employers such as flexible working arrangements or working remotely, given that families of children with LSDs are low-income families particularly vulnerable to negative economic consequences (lay-offs and reduction in income) of the pandemic.

## What are the LSD-specific and COVID-19-based optimal care patterns during pandemic?

The management of children with LSD during the pandemic is challenging in terms of continued treatment of LSD during the time of crisis and treatment of infection in the presence of LSD. The COVID-19 pandemic significantly affects the LSD community by creating additional anxiety and fear of being infected with the virus both during the journey to hospital centers for therapy and during the therapy itself (62). Along with limited numbers of referral centers and experts in specialized care because of reorganization, this is likely to result in treatment interruptions, particularly for in-hospital ERT infusions.

Accordingly, in a phone questionnaire survey of 102 Italian patients with LSDs (mean age 38.8 ± 18.6 years, 51% were males) including Gaucher disease (n = 44), Pompe disease (n = 16), Fabry disease (n = 15), MPS (n = 12), Niemann Pick disease type C (n = 10) and cystinosis (n = 5), authors reported that none of the patients had proved COVID-19 or experienced problems in receiving oral treatments but disruptions occurred in ERT (63). Specifically, while 77.5% of patients were on in-hospital infusion therapy and 22.5% were on home-therapy before COVID-19 outbreak, 49% and 6% of in-hospital and at-home treated patients reported interruptions, respectively; due to fear of infection (62.9%) and reorganization of the infusion centers (37%) in most cases (63). Authors considered the home-infusions as the most efficient way to maintain therapy access during pandemic provided that the personnel involved is monitored and adhere to the correct use of PPE (63).

In a very recent paper, E-IMD consortium reported the results of a survey done with healthcare providers following-up 792 patients with intoxication type inborn metabolic diseases. The results of the survey revealed that there was an increased demand for medical services most of which was covered by remote technologies. The follow-up visits were noticed to be reduced by 41%. Most of the infected patients (83%) showed mild clinical symptoms. The survival rate during the study period was reported to be 100% (64).

MetabERN collaboration group investigated the incidence of COVID-19 in IMD patients during the early phase of the pandemic (March and April 2020). This study involved two surveys; one for the patient organizations and the second for healthcare providers. In the second survey, 52% of the healthcare providers stated that patients with LSDs are expected to be at major risk for COVID-19. Also in this paper, it is stated that patients with inborn errors of metabolism are at risk for not being able to receive their routine health service during the pandemic (65).

A recent paper by Paneghetti has given the results of an online survey regarding SARS-CoV-2 infection rates and severity of symptoms for patients with inborn errors of metabolism followed-by MetabERN healthcare providers. The overall prevalence was 1716/100.000 (452 infected patients out of 26,347). It was also noted that 38.2% of the pediatric patients and 69.2% of the adult patients who were tested positive for COVID-19 had LSDs (66).

Ramaswami et al. investigated the effects of COVID-19 pandemic on patients with LSDs in United Kingdom. Based on the recommendations in United Kingdom, the authors defined the situations according to which LSDs should be clinically prioritized. For example, MPSs patients with spinal and airway problems are recommended to have an emergency approach (67).

Data from National Organization for Rare Disorders (NORD®) COVID-19 Community Survey Report on the critical issues and concerns faced by the rare disease community during pandemic among 772 participants (72% have a rare disease, others responding as a caregiver or family member, 75% aged over 40 years) revealed that 95% of respondents are affected by the pandemic in terms of immediate, long-term health and wellbeing, 98% are worried about COVID-19 (67% are very or extremely worried), 74% have had a medical appointment canceled (65% were offered an alternative appointment *via* telephone or video) and 69% of respondents are concerned about medication and medical supply shortages (68).

However, if certain measures are taken enough to convince the patients and families about low risk of infection during treatment, compliance is significantly increased. For example, 88 of 104 patients with LSD being followed up in a referral center for metabolic diseases in Turkey were phone-interviewed to analyze the impact of the pandemic on ERT implementation, and 13 (14.7%) patients reported experience of interruptions due to fear of infection, while after phone-interview only 5 of these families agreed to visit the hospital to receive ERT and 8 families remained staying at home since they are extremely worried (*unpublished communication*).

The likelihood of experiencing ERT interruptions during COVID-19 pandemic could lead to deterioration in renal functions in patients with Fabry disease for example who were treated with reduced dosage of enzyme because of the shortage of agalsidase-β supply between 2009 and 2012 (63, 68). It was previously observed that interruption of treatment in patients with Gaucher disease resulted in worsening organomegaly as well as deterioration in hematological parameters (69). Also, there is data about the effects of ERT interruption in MPS and Pompe diseases which point out to worsening of visceromegaly, respiratory function and walking capacity for the former (70–74) and to irreversible clinical decline for the latter (75). Hence, the continuation of ERT in Gaucher; Fabry, and Pompe diseases as well as mucopolysaccharidosis without prolonged interruption during pandemic even for infected cases seems very important for the long-term outcome.

Hence, the real risk of suspending ERT, even if for short periods during COVID-19 pandemic, should carefully be considered given that the cumulative doses of ERT enable improved clinical outcome and the worsening of disease is possible if ERT is discontinued even on a monthly basis. In addition, there are concerns regarding the likelihood of certain treatments (i.e., immunosuppressive therapy in Pompe disease) to render them more vulnerable to infections (11).

Another potential challenge seems to be the safe use of medicines to treat COVID-19 in case of an infection, given the likelihood of adverse events (11).

### Consensus statements on LSD-specific practice patterns

Due to concerns regarding the increased risk of SARS-CoV2 infections and severe disease in children with Pompe disease receiving immunosuppressive treatment because of antidrug antibodies or allergic reactions, many patients have the tendency to stop their immunosuppressive treatments. However, it is important to note that many commonly used immunosuppressive drugs such as JAK kinase inhibitors and tocilizumab have even been proposed and/or used for the treatment of selected patients who develop cytokine storm following COVID-19 infection (76). Current WHO guideline recommends antivirals, immunomodulators and other adjunctive therapies including hydrochloroquine for children with COVID-19 should be administered in the context of clinical trials. Because high-quality evidence has not been established in the studies of these drugs and significant adverse effects have also been observed (77). European Pediatric Rheumatology Association also recommended continuing methotrexate, biologics and low dose corticosteroids for rheumatic diseases during pandemic (78). For those reasons the members of the Adboard recommended to continue immune modulation treatment for patients with Pompe disease without COVID-19 if strict measures to prevent infection such as isolation, physical (social) distance, and good hygiene are provided. But if the patient with LSD has active COVID-19 infection, the final decision should be given by the metabolic physician and pediatric infectious specialist based on the clinical progression.

Accordingly, the continuation of ongoing immunomodulatory and/or ERT in children with LSDs necessitates the referral centers to ensure the administration of the therapy safely with effective measures or precautions. Currently, this is provided *via* a pre-triage algorithm on patient admission to discriminate and separately manage normal vs. suspected COVID-19 cases in some hospitals, while the physical capacities of the hospitals play a crucial role in the quality and/or applicability of these measures. In addition, patients in Turkey are well-informed and strictly adhere to all the necessary precautions and safeguards to prevent being infected both before and after the therapy. Nonetheless, while we encourage families to continue therapy through e-mail or phone consultations or *via* Instagram posts for any questions or concerns, they might have, the final decision whether or not to come to the hospital for infusions is at family's discretion.

Disruptions in therapy are likely for families with fear of infection and extreme anxiety regarding getting an infection during public transport or in-hospital intervention. This seems to indicate the likelihood of home infusions to offer a convenient solution for children with LSDs in this crisis time, particularly for those families who prefer temporary discontinuation of therapy to the risk of infection. However, home infusions for LSDs are not could not be provided to the patients in Turkey because of the lack of relevant regulations. But it is out of the question how useful home infusions would be during times of pandemic or other disasters provided that adequate healthcare personnel (i.e., medical doctor, nurse) and equipment (i.e., monitors and defibrillators) would be present throughout the infusion.

Hence, it seems necessary that frequent and continuing contacts and meetings with the health authorities to provide information on special needs and priorities of children with rare diseases in the pandemic period and to form the regulatory basis of home infusions in this patient population should be considered.

Creation of SARS-CoV2 free hospitals and totally pandemic hospitals, local infection-free outpatient clinics serving specifically for children under infusion therapy and availability of home infusions seem to be potential measures in fighting with pandemic without compromising the care of children with LSD while improving existing infrastructure and adapting continuous implementation of effective measures or precautions locally in each center to ensure the management of children safely seem to be a more realistic option.

### Consensus statements on COVID-19-based practice patterns

For LSDs patients with exposure to SARS-CoV-2 infection, to maintain close communication with a metabolic and infectious diseases specialist is mandatory, given that acute care providers or physicians from other specialty/subspecialties would not be familiar with critical aspects of LSDs, interfering with optimal care (23).

It is a prudent clinical practice to adopt a preemptive approach to screen and diagnose SARS-CoV-2 in patients with LSDs based on exposure history and/or early disease symptoms, monitoring and proactive management of SARS-CoV-2 infection in children with LSDs are imperatives given their potential risk of developing severe COVID-19 (23).

It should be noted that the laboratory tests such as ferritin, CRP, sedimentation rate, blood counts, liver function tests, and D-dimers used to estimate progression in COVID-19 patients, may already be elevated in patients with LSD regardless of COVID-19. For this reason, it is important to consider individual pre-pandemic levels of these markers as the baseline in these patients and provide a higher level of care in those with elevated biomarkers.

Currently applied treatment protocols in COVID-19 infected adult patients are based on previous studies of SARS CoV-2 at genome level and involve hydroxychloroquine/ chloroquine with or without azithromycin, targeted immune-suppressants such as inhibitors of IL-6 signaling (e.g., Tocilizumab, Sarilumab), TNF-a signaling (e.g., Humira), IL-1b signaling (e.g., Anakinra) and Janus kinase (JAK) inhibitors (e.g., Ruxolitinib, Baricitinib, Tofacitinib) as well as protease inhibitor lopinavir/ritonavir (e.g., Kaletra) and COVID-19 convalescent plasma (26, 27, 79). Selection of antiviral treatment in children is applied considering the clinical condition of the disease and the underlying clinical disease that may increase the risk of progression. There is no proven treatment for COVID-19 in children. Antivirals should be used in the context of clinical trials in children.

Important considerations in children with LSDs are drug-drug interactions with current empirical therapies for SARS-CoV-2 infection, including hydroxychloroquine, antibiotics (such as azithromycin) and antiviral agents (23). Similar to hydroxychloroquine and azithromycin, eliglustat has been shown to prolong QTc intervals at supra-therapeutic doses (23). Hence, 'prophylactic' use of hydroxychloroquine, with or without azithromycin in patients with LSDs receiving certain medications might require additional caution, while it may also be prudent to interrupt eliglustat treatment when specific therapies for SARS-CoV-2 infection known to cause prolonged QTc is initiated (23). Additionally, current literature does not recommend use of hydroxychloroquine in combination with azithromycin in treatment of children with COVID-19 (77, 80).

Regardless of the reason and clinic for admission, each child with LSDs should be consulted with pediatric metabolic disease specialist and or expert center to understand the specific disease history and treatment plan of the patient.

## Conclusions

The COVID-19 pandemic has introduced many challenges related to the treatment and support of patients with LSDs. It seems that interestingly some common pathophysiological mechanisms exist there between LSDs and COVID-19 regarding lysosomal involvement or disruption, immune-inflammatory mechanisms and even end-organ damage and prognostic biomarkers in some diseases. But of course, further studies are needed to clarify certain aspects related to immunity, lysosomal dysfunction and disease pathogenesis. The continuation of in-hospital treatment on a regular basis seems to be the most challenging factor in the management of children with LSDs during COVID-19 pandemic, given the unavailability of home infusions and extreme worries and fear of infection among families about in-hospital infusion therapy, despite they refer to a population with strict adherence to preventive measures. Hence, given the significant risk of treatment interruption in worsening of clinical status and disease progression, increase the susceptibility and poor prognosis of infection, every effort should be made to enable LSD patients to continue their therapy. Collaboration with family groups and telemedicine for follow ups during the pandemics seem also important. There is a need for nationwide cross-sectional screening study to evaluate the healthcare resource gaps and therapy disruptions in LSD community during pandemic as well as the socioeconomic and psychological impacts to ensure optimal health care during pandemic. In fact, a national registry seems to be necessary to better understand the pattern and natural history of SARS-CoV-2 infection in children with LSDs providing effective preventive measures and treatment policies.

## Author contributions

All authors contributed equally to the conception of this review, the search methodology, and the writing of this manuscript. All authors critically revised the manuscript, contributed to the discussion, read, and approved the final manuscript.
